# Efavirenz and Lopinavir/Ritonavir Alter Cell Cycle Regulation in Lung Cancer

**DOI:** 10.3389/fonc.2020.01693

**Published:** 2020-08-28

**Authors:** Rahaba Marima, Rodney Hull, Zodwa Dlamini, Clement Penny

**Affiliations:** ^1^SA-MRC/UP Precision Prevention and Novel Drug Targets for HIV-Associated Cancers Extramural Unit, Faculty of Health Sciences, Pan African Cancer Research Institute, University of Pretoria, Pretoria, South Africa; ^2^Department of Internal Medicine, Faculty of Health Sciences, School of Clinical Medicine, University of the Witwatersrand, Parktown, South Africa

**Keywords:** efavirenz, lopinavir/ritonavir, cell cycle, lung cancer, cell proliferation, cell death, real-time cell analysis

## Abstract

Highly active anti-retroviral treatment (HAART) is currently the most effective treatment for HIV/AIDS. Additionally, HIV positive patients receiving HAART have a better health-related quality of life (HRQoL). Cancers previously associated with HIV/AIDS also known as the AIDS defining cancers (ADCs), such as Kaposi's sarcoma and non-Hodgkin's lymphoma have been on the decline since the introduction of HAART. However, non-AIDS defining cancers (NADCs), in particular, lung cancers have been documented to be on the rise. The association between the use of HAART components and lung carcinogenesis is poorly understood. This study aimed at elucidating the effects of two HAART components [efavirenz (EFV), and lopinavir/ritonavir (LPV/r)] on lung cancer. This was achieved through the use of *in vitro* cell biological approaches to assess cell health, including cell viability, Real Time Cell Analysis (RTCA) growth monitoring, evaluation of the cell cycle, and progression to apoptosis, following on drug treatments. At plasma level concentrations, both EFV and LPV/r induced S-phase arrest, while at lower concentrations both drugs promoted the progression of cells into G2/M phase following cell cycle FACS analysis. At higher concentrations although cell viability assays reflected anti-proliferative effects of the drugs, this was not statistically significant. RTCA showed a significant decline in cell viability in response to the highest dose of LPV/r. Dual staining by Annexin V-FITC and PI confirmed significant pro-apoptotic effects were promoted by LPV/r. Both EFV and LPV/r exert double-edged oncogenic effects on MRC-5 and A549 lung cells, acting to either promote cell proliferation or to enhance apoptosis. This is affected by EFV and LPV/r altering cell cycle progression, with a significant S-phase arrest, this being an indication of cellular stress, cytotoxicity, and DNA damage within the cell.

## Introduction

HIV infection is a major global concern with increasing prevalence. In 2018, UNAIDS estimated that ~37.9 million people were living with HIV, 1.7 million people were newly infected, while ~0.77 million people died from AIDS-related illness. An estimated 23.3 million people were receiving antiretroviral treatment (ART) (1). In total, an estimated 32 million people have died of the disease since the first cases of AIDS were reported in 1981. Long term effects of HAART exposure on cancer risk are not well-defined. In this regard according to basic and epidemiological research, there might be specific associations of each HAART component with distinct patterns of cancer risk (2). Currently, the human immunodeficiency virus acquired immunodeficiency syndrome (HIV/AIDS) and lung cancer are arising as colliding epidemics and urgent interventions are necessary to combat these leading causes of morbidity and mortality (3). In addition, cancer incidence rates are also shown to be increased in people living with HIV/AIDS (PLWA) compared to the general population (4–7). To date, there is no cure for HIV/AIDS and highly active antiretroviral treatment (HAART) is the most effective treatment regimen (8). Additionally, there has been a decline in cancers previously associated with HIV/AIDS, also known as the AIDS defining cancers (ADCs): including Kaposi's sarcoma, primary central nervous system lymphoma, non-Hodgkin's lymphoma, and cervical cancer. In contrast to this, non-ADCs have been documented to be on the rise in the HAART era, with lung cancer emerging as a leading NADC (6, 9).

Lung cancer is one of the leading NADCs both globally and in South Africa (10). In South Africa, adenocarcinoma is the most common form of lung cancer (10–12). Lung cancer is characterized by high genetic diversity (13). Genetic mutations in Ki-ras2 Kirsten rat sarcoma viral oncogene homolog (KRAS), epidermal growth factor receptor (EGFR), B-RAF (BRAF), and phosphatidylinositol 3-kinase (PI3K) signaling oncogenic pathways have been identified in lung cancer. The aberrant expression of TP53, PTEN, RB1, LKB11, and p16 tumor suppressor genes in lung cancer has also been reported. Other gene targets with genetic alterations in lung cancer include human epidermal growth factor receptor (HER2), Mitogen-activated protein kinase (MEK), Anaplastic lymphoma kinase (ALK), (ROS1) and Fibroblast growth factor receptor 1 (FGFR1) (14–17). Smoking remains one of the significant factors in lung carcinogenesis (16). However, the association between lung cancer and the use of HAART components is poorly understood. The identification of genetic markers in the development and progression of lung cancer has made significant improvements in the understanding of lung cancer molecular pathogenesis and overall patient diagnosis and treatment. In addition, when compared to the same age group in the general population, the risk of developing non-small cell lung carcinoma (NSCLC), the most predominant form of lung cancer, is higher in HIV positive patients (10). While South Africa has the largest HIV epidemic and antiretroviral therapy (ART) program in the world (18, 19), the poor understanding of the relationship between the use of HAART components and tumorigenesis especially lung cancer has placed a burden on public health, globally and in South Africa. This study aimed at determining the effects of two HAART components (EFV and LPV/r) on lung cancer. Cell viability, cytotoxicity assays, cell cycle analysis, and apoptosis assay were performed on treated MRC-5 and A549 cells. Treatment with EFV and LPV/r alters the cell cycle progression, with a significant S-phase arrest, cellular stress, DNA damage, and cytotoxicity.

## Materials and Methods

### ARV Drugs

The ARV drugs for this study were purchased from Toronto Research Chemicals (Toronto, Ontario, Canada), and prepared as stock solutions in pharmaceutical/analytical grade methanol. The mean steady-state peak plasma concentration (Cmax) is the most physiologically relevant concentration for the ARVs because it represents naturally occurring concentration of the drugs following their intake (20). The concentrations used in this study includes the clinically relevant plasma level doses and experimental doses.

### Cell Culture

The lung cell lines MRC-5, normal lung fibroblast (ATCC CCL171) and A549, lung adenocarcinoma (ATCC CCL185) were obtained from the American Type Culture Collection (ATCC). MRC-5 and A549 cells were grown in Dulbecco's Modified Eagle Medium (DMEM, Life Technologies, Inc, Rockville, MD) supplemented with 10% heat-inactivated fetal bovine serum (Sigma-Aldrich, St. Louis, MO) and 1% penicillin and streptomycin (GIBCO). Cells were cultured in 25 cm^2^ cell culture flasks (Corning, USA) and were kept in a CO_2_ incubator at 37°C in a humidified atmosphere with 5% CO_2_ in air. For experimental purposes, cells cultured to an exponential growth phase (at ~70% confluency) were used. Cells were then serum-starved for 24 h to synchronize the cell cycle. The following day, the cells were pharmacologically treated with either EFV at concentrations of 4, 13, 26, or 50 μM, respectively; or with LPV/r at concentrations of 10, 32, 50, or 80 μM, respectively. Treatment was carried out for 24–72 h. Control cells were exposed to growth medium and vehicle only (methanol 0.1% v/v).

### Alamar Blue (AB) Cell Viability Assay

The Alamar Blue (AB) cell viability assay was used to measure MRC-5 and A549 cell viability in response to EFV and LPV/r treatment, respectively, and relative to (0.1v/v) methanol, the vehicle control. Confluent cells were trypsinised and harvested by centrifuging; the cell pellets were re-suspended in a small volume of cell culture medium. An aliquot of cells was then counted using an automated cell counter (Bio-Rad) and 2 × 10^3^ cells were seeded in a 96-well-plate). Cells were allowed to attach and grow overnight. Prior to treatment, the cells were serum starved for 24 h to synchronize the cell-cycle. The cells were treated in triplicate with one of the following treatments: a vehicle control consisting of 0.1%; v/v methanol; one of four different concentrations of EFV (4, 13, 26, 50 μM), respectively; and one of four different concentrations of LPV/r (10, 32, 50, 80 μM), respectively. Treatment time was for a period of either 24, 48, or 72 h, respectively. At the end of each treatment phase, AB was added directly into culture media in each well at a final concentration of 10% and incubated for 3–4 h at 37°C in an atmosphere of 5% C0_2_ in air. The absorbance of test and control wells was measured at 540 and 630 nm, wherein the number of viable cells correlates with the magnitude of dye reduction and is expressed as percentage of AB reduction (21). The calculation of the percentage of AB reduction (%AB reduction) is as follows, according to the protocol reduced controls are:

$$\begin{array}{l} {\text{\%Reduction}=\frac{\varepsilon \text{oxid }630\text{ nm (sample A}450\text{nm)}-\varepsilon \text{oxid }540\text{ nm (sample A}630\text{ nm)}}{\varepsilon \text{red}540\text{ nm(oxidized control A}630\text{ nm)}-\varepsilon \text{red}630\text{ nm (oxidized control A}450\text{nm)}}\}}_{\times 100} \end{array}$$

The molar extinction coefficients of AB for the oxidized and reduced controls are:

εoxid 630 nm = 34.798, εoxid 540 nm = 47.619, εred 630 nm = 5.494, and εred 540 nm =104.395 (22).

The values of % AB reduction was corrected for background values of blank wells containing AB and medium only without cells. The % AB reduced corresponded to the percentage of viable cells and was a functional indicator of cell viability in response to ARV drug treatment over 24–72 h.

### xCELLigence RTCA Cell Proliferation and Cytotoxicity Assay

Cell proliferation was measured using the xCELLigence Real-Time Cellular Analysis (RTCA) system (ACEA Biosciences), which allows researchers to monitor the cell viability and cell growth continuously at multiple time points. Cells were seeded at a density of 1 × 10^4^ cells per well of the 16-well E-Plate and this was placed on the docking station contained within the incubator. The cells were then left to grow for 24 h with the RTCA instrument taking readings every minute. Following this, cells were treated with EFV (4, 13, 50 μM) or LPV/r (10, 32, 80 μM). A vehicle control consisted of 0.1% v/v methanol. During the treatment phase, the cells were continuously monitored for up to 100 h, with a reading being taken every 15 min. Cell sensor impedance was expressed as an arbitrary unit termed the Cell Index (CI). To eliminate variation between wells, the cell index values were normalized to the value at the beginning of treatment time-point; and thus, a normalized cell index (NCI) was used to determine cell viability.

### Cell-Cycle Analysis by FACS

Analysis of the cell cycle distribution in response to ARV treatment was performed by seeding 1 × 10^5^ cells/ml overnight in 25 cm^2^ flasks and treating them with one of four different concentrations of EFV (4, 13, 50 μM), or with one of four concentrations of LPV/r (10, 32, 80 μM) for 24–48 h. After treatment cells were fixed with 70% ethanol at −20°C for 1 h. Next, cells were washed twice with PBS, treated with 10 mg/ml RNAse (Sigma) and stained with 25 μl of PI (1 mg/ml), (Sigma) and incubated at 4°C overnight in the dark. All experiments were performed in triplicate. The stained cells were analyzed on the BD Accuri C6 FACS instrument and results were generated and analyzed as histograms (G1, S, and G2 phases) using the BD C6 Accuri software.

### Apoptosis Assay Using Annexin V-FITC and Propidium Iodide (PI) Dual Staining

In order to carry out an apoptosis assay by flow cytometry, MRC-5 and A549 cells were seeded at a density of 1 × 10^5^/ml in 25 cm^2^ flask overnight before being treated LPV/r at various concentrations for 24–48 h. Camptothecin (CPT) (50 μM) (Sigma) treatment was used as a positive control to induce apoptosis. Determination of apoptotic cell numbers by fluorescent staining was done using the Annexin V FITC/PI apoptosis kit from Santa Cruz Biotechnology, following manufacturer's instructions. Briefly, cells were incubated in triplicate with Annexin V FITC and propidium iodide (PI) in binding buffer for 15 min in the dark; and stained cells were immediately subjected to flow cytometry analyses using the BD C6 Accuri flow cytometer (BD Biosciences).

### Statistical Analysis

Results for this study were analyzed using Graph-Pad Prism 5 and expressed as means ± standard error of the mean (SEM). Significant differences were determined using one-way analysis of variance (ANOVA) followed by Tukey's *post-hoc* test. A probability level of *p* < 0.05 was considered significant.

## Results

### Alamar Blue Assay, Figure 1

The physiological reduction of the Alamar Blue (AB) dye was used here to quantitatively measure both cell proliferation and viability of MRC-5 and A549 cells in either EFV or LPV/r treated and vehicle control cells.

**Figure 1:**
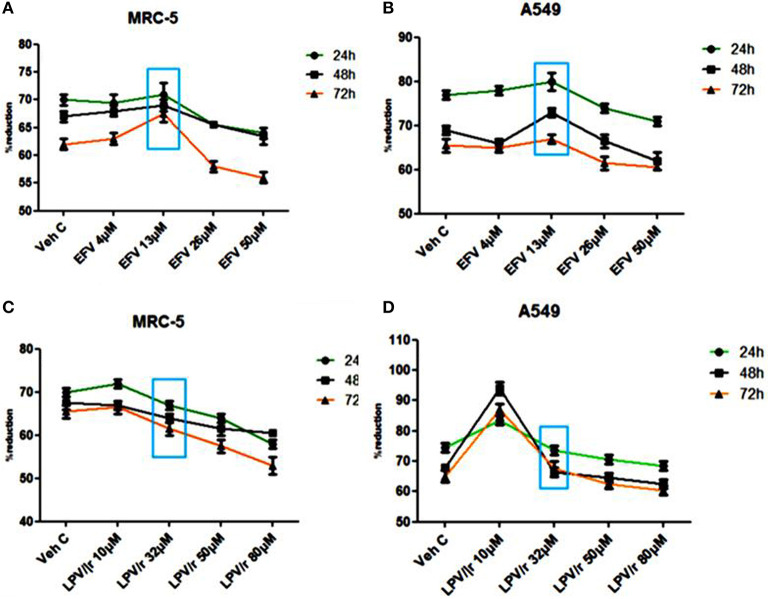
Alamar blue assay analysis. **(A)** The percentage (%) of AB reduction representing the MRC-5 cell viability. **(B)** The A549 cell viability in response to the EFV drug treatment. **(C)** The MRC-5 cell viability in response to LPV/r drug treatment relative to control. **(D)** The representation of the A549 cell viability in response to the LPV/r cytotoxic effects. A–D represent treatments vs. control at 24, 48, and 72 h, the blue box indicates the most relevant physiological dose, and effects on cell viability are statistically insignificant, with *p* > 0.0.5. The graphs are a representative of three independent experiments, which were done in triplicate each.

#### Efavirenz (EFV) Treatment, Figures 1A,B

The reduction of AB was monitored at 24 h intervals (24, 48, and 72 h) and measured spectrophotometrically at 540 and 630 nm. Figures 1A,B illustrate the percentage reduction of AB by MRC-5 and A549 cells in response to EFV, respectively. As represented in Figure 1, 4 μM EFV did not significantly change cell viability over a 24–72 h treatment period. At 13 μM (physiological dose and indicated by the blue box), the slight increase in cell proliferation at all three-time intervals was not significant. Similarly, a decline in cell proliferation with 26 and 50 μM treatment was also not significant.

#### Lopinavir/Ritonavir (LPV/r) Treatment, Figures 1C,D

Cell proliferation and viability following LPV/r treatment is shown in Figures 1C,D for the MRC-5 and A549 cell lines, respectively. When compared to the control cells, the 10 μM LPV/r treatment, was shown to have insignificantly increased proliferation, while at 32 μM there was a slight but insignificant decrease in proliferation. Concentrations of 50 and 80 μM LPV/r, decreased MRC-5 cell viability (see Figure 1C), but these effects of LPV/r on MRC-5 cell viability were not significant. A change in AB% reduction in A549 cells was observed following treatment with a range of LPV/r concentrations: at 10 μM LPV/r, the cells proliferated relative to the vehicle control cells. A decline in AB% reduction occurred with the 32 μM LPV/r treatment at all three time points. While treatment with both 50 and 80 μM LPV/r had an anti-proliferative effect on the A549 cells, the observed changes were however statistically not significant.

### Real-Time Cell Analysis (RTCA) of Cytotoxicity Using xCELLigence, Figure 2

The potential cytotoxic effects of EFV on the MRC-5 and A549 cells were determined by plotting the growth curves acquired as a function of cell index (normalized to 1) vs. time (h) over a period of ~100 h. Since the cell index is proportional to cell viability, the greater the cell index, the better the cell viability. Based on the preceding AB data, three of the four ARV concentrations were further selected for the cytotoxic, cell viability, and proliferation assays using RTCA. For these evaluations, both cell lines were treated with one of three concentrations of EFV (4, 13, 50 μM), respectively; or one of three concentrations of LPV/r (10, 32, 80 μM), respectively. To further analyse the effects of EFV and LPV/r on cell proliferation in a time dependent manner, the slope function of the curve was used. This function describes the steepness, incline, gradient, or changing rate of a curve within the given time period; and provides a measure for parameters of cell proliferation, cell adhesion, receptor activation, cytotoxicity, and other indicators of cell behavior. Here, the slope function was used to determine the rate of change of the cell index (CI) or normalized cell index (NCI) for the cells following drug treatment.

**Figure 2:**
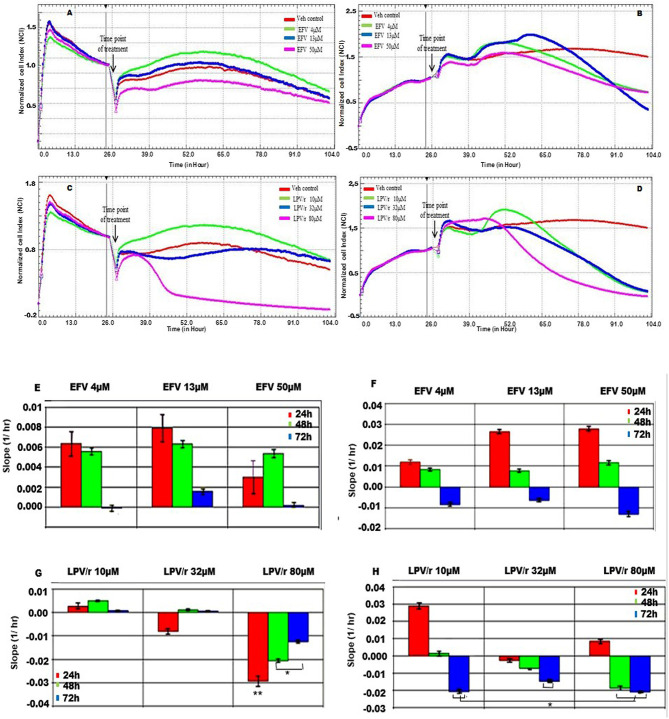
MRC-5 and A549 cell proliferation in response to EFV and LPV/r. **(A)** Cell growth curve of MRC-5 cells treated with EFV **(B)** Growth curves representative of A549 cells treated with EFV. **(C)** MRC-5 growth curves representing cells treated with LPV/r. **(D)** Growth curves for A549 cells treated with LPV/r. The curves were plotted as a function of normalized CI vs. time in ARV treated vs. control. **(E)** The slope function of MRC-5 cells representing the response to EFV treatment over a 24 h time. **(F)** The slope function representing the response of A549 cells to EFV drug treatment at 24 h time intervals. **(G)** The slope function demonstrating MRC-5 cell response to LPV/r drug treatment, monitored at 24 h intervals. **(H)** A slope function representing A549 cells treated with LPV/r at 24 h intervals. The slope function represents the rate of cell detachment, and thus cell death for each of the drug concentrations. Results represent three independent experiments done in triplicate each. Error bars denote SEM; *p < 0.05; **p < 0.01.

#### EFV Treatment Response in MRC5, Figures 2A,E

With reference to Figure 2A, following treatment at 24 h, all MRC5 cells whether treated with either EFV or with methanol (vehicle control) they continued to proliferate. Additionally, cells treated either with 4 μM or 13 μM EFV proliferated more than the vehicle control cells. In contrast to this, cells treated with 50 μM EFV had a decreased cell proliferation. The slope function of MRC-5 cells treated with either 4 μM or 13 μM EFV, indicated an increase in cell proliferation and growth after 24 h of treatment (Figure 2E). A steady decline in cell proliferation was noted at 48 h, this being more evident at 72 h, indicating the onset cell detachment/cell death. Treatment of MRC-5 cells with 50 μM EFV resulted in a slight increase in cell proliferation and growth at 24 h, with further growth at 48 h; followed by a steep decline in cell proliferation at 72 h.

#### A549 Cell Response to EFV, Figures 2B,F

After 24 h of exposure to the vehicle control and lower concentrations of EFV, A549 cells grew and proliferated steadily. At 48 h after treatment the vehicle control continued to proliferate steadily, while the cells treated with 4 and 13 μM EFV showed a decrease in cell viability (Figure 2B). Cells treated with 50 μM EFV proliferated slowly compared to the vehicle control cells, indicating an anti-proliferative effect of 50 μM EFV on the A549 cells. The slope function plot for cell response to EFV reflected a slight increase in cell proliferation for 4, 13, and 50 μM, respectively, after 24 h of treatment (Figure 2F). At 48 h there was decreased cell proliferation, with a marked decline at 72 h in cell viability for each of the three drug concentrations.

#### MRC-5 Cell Response to LPV/r, Figures 2C,G

The MRC-5 vehicle control cells continued to grow and proliferate steadily. In comparison, MRC-5 cells treated with 10 μM LPV/r increased in proliferation compared to the vehicle control (Figure 2C). However, at a concentration of 32 μM LPV/r the cells neither increased nor decreased their proliferation, which indicated cell-cycle arrest. In contrast to this, treatment of MRC-5 cells with 80 μM LPV/r was clearly cytotoxic to the cells, indicated by an abrupt peak of the normalized CI immediately after drug treatment, followed by a rapid decline in cell viability. The slope-function plot reflected the growth trends of the real time growth curves (Figure 2G). At a concentration of 10 μM LPV/r the cells continued to grow progressively for 24 and 48 h after treatment, followed by a decline in cell viability after 72 h.When the cells were treated with 32 μM of LPV/r, there was a slight decrease in cell viability 24 h after treatment, followed by a slight increase in cell proliferation at 48 h; and this remained steady even after 72 h of drug exposure. At 24 h following 80 μM LPV/r treatment, there was a marked decline in cell viability. This decrease in cell viability persisted at 48 and 72 h after treatment.

#### A549 Cell Response to LPV/r, Figures 2D,H

The A549 cells were monitored before and after drug treatments at 24 h post seeding (refer to Figure 2D). When compared to the control cells, A549 cells treated with 10 μM and 80 μM LPV/r showed a proliferative effect, followed by a rapid decline in cell viability. The 32 μM treated cells in contrast, displayed a cell-cycle arrest (observed from the time point of treatment), after which there was a decrease in cell viability. The slope function plot for cell response to LPV/r revealed an apparent increase in A549 cell proliferation for cells treated with 10 μM LPV/r at 24 h, while there was a decline in cell viability when cells were treated with 32 μM LPV/r at 24 h. This steady decrease in cell viability for cells treated with 32 μM remained consistent even after periods of 48 and 72 h. There was an initial increase in proliferation for cells treated with 80 μM LPV/r (Figure 2H), followed by an abrupt decline in cell viability at 48 and 72 h.

#### RTCA Demonstrates the Pro-and-Anti-proliferative Effects of EFV and LPV/r

The label free RTCA assay was particularly sensitive to and indicative of the window period of the drug efficacy. This was reflected by the growth curves and further analyzed by the slope function, showing the associated decline in CI, and therefore in cell viability. At lower concentrations EFV had the effect of stimulating cell proliferation in both the MRC-5 and A549 cells, relative to vehicle control cells. Subsequently, proliferation (CI) decreased at higher concentrations with the occurrence of cellular detachment from the culture substrate. However, while there was no clear distinction here in the growth and proliferation patterns between treated and vehicle control cells, there were nevertheless differences observed in the decreases and increases in the proliferation rates between the vehicle control and treated cells. This finding suggests that although EFV treatment does seem to influence cell proliferation, it may not necessarily alter cellular health. Similar to EFV, LPV/r at low concentrations stimulated cell proliferation in both MRC-5 cells and excessively so in A549 cells, followed by cell death. An intermediate dose, caused cell-cycle arrest in both cell types, while high concentrations led to a significant increase in cell death, preceded by increased cell proliferation.

### Cell-Cycle Analysis by FACS, Figure 3

**Figure 3:**
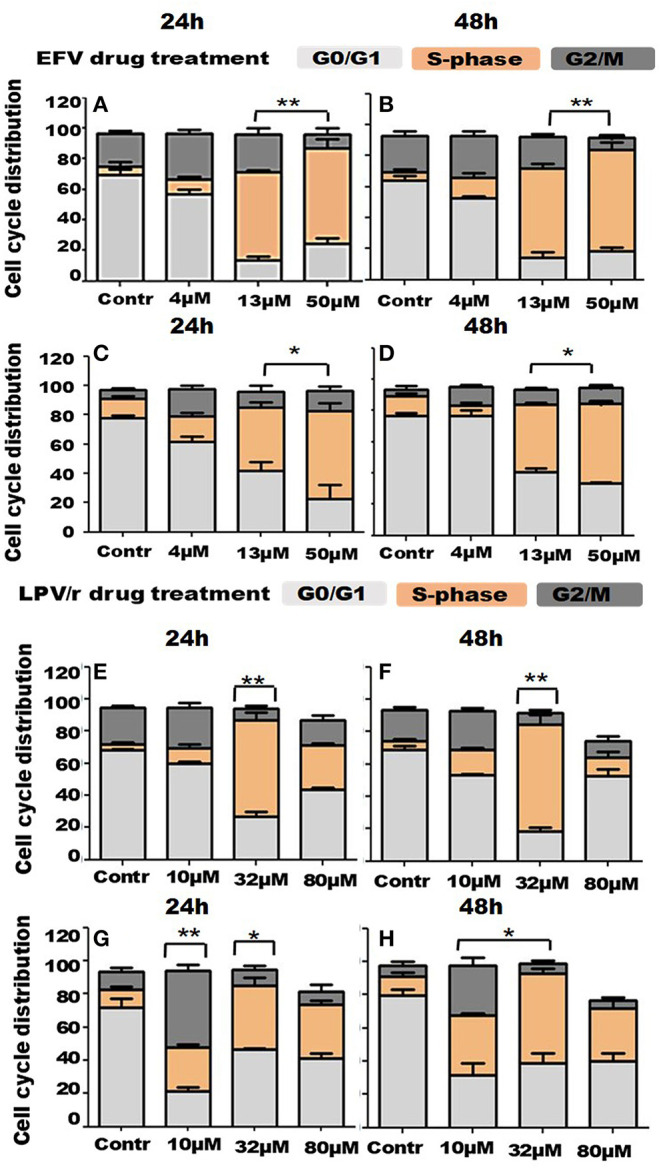
Cell cycle distribution in response to ARV drugs. **(A,B)** Show bar graphs of EFV treated MRC-5 cells at 24 and 48 h. **(C,D)** Demonstrate bar graphs of A549 cells treated with EFV at 24 and 48 h. **(E,F)** Illustrate bar graphs of MRC-5 cells treated with LPV/r at 24 and 48 h, while **(G,H)** are bar graphs of A549 LPV/r treated cells at 24 and 48 h. The increase in S-phase, (S-phase arrest) observed here is statistically significant with *p* < 0.0 1. A significant increase in G2/M with *p* < 0.05 in A549 10 μM LPV/r treated cells is also evident. Error bars denote SEM; *p < 0.05; **p < 0.01.

Since RTCA analysis demonstrated some effects of EFV and LPV/r on the cell-cycle, flow cytometry was employed to quantify DNA content and thus the particular stage of the cell-cycle treated cells were in, relative to the vehicle control cells. Here, the scope of this analysis was to determine the regulatory effects of ARV's on the cell-cycle in lung cells. Prior to drug treatment and cell-cycle analysis, cells were serum-starved overnight to synchronize the cell-cycle at G0/G1. Results are a represented in Figure 3 as bar graphs, where data is expressed as mean ± standard error of the mean (SEM).

#### FACS Analysis of EFV Treated MRC-5 Cells

About 73% of the vehicle control cells were located in the G0/G1 phase of the cell-cycle, at both 24 and 48 h. Relative to this, the percentage of cells in G0/G1 decreased with increased drug concentration at 24h, decreasing to about 60% (4 μM), 10.5% (13 μM), and 21% at 50 μM. At 48 h however, 54% cells treated with 4 μM were in G0/G1, before decreasing to 10% (13 μM) and 16.4% (50 μM). In association with this, the percentage of cells undergoing DNA synthesis in S-phase, began to significantly increase, from 3% in (normal) control cells, to 10% (4 μM), 60% (13 μM), and peaking at about 70% (50 μM), at both the 24 and 48 h time points. While about 20% of control cells were in G2/M, this percentage increased to ~28–30% when cells were treated with 4 μM EFV; and decreased again to 18–19% of cells treated with 13 μM EFV; and further to about 5–6% of cells following treatment with 50 μM EFV (see Figures 3A,B).

#### FACS Analysis of EFV Treated A549 Cells

Approximately 80% of the vehicle control cells were located in the G0/G1 phase of the cell-cycle, at both 24 and 48 h. Relative to this, the percentage of cells in G0/G1 decreased with increased drug concentration at 24 h, reducing to about 58% when treated with 4 μM EFV, 33% when treated with 13 μM EFV and 13% when treated with 50 μM EFV. At 48 h however, 80% of cells treated with 4 μM EFV remained in the G0/G1 stage, before decreasing to 30% (13 μM) and 22% (50 μM). In relation to this, a significant increase in the proportion of cells in S-phase with increasing EFV dose was observed. This S-phase increase ranges from 11% in vehicle control cells, to 15, 40, and 55% in cells treated with 4, 13, and 50 μM treatment with EFV at 24 h. This trend was also noted at 48 h, when cells were treated with 4 μM EFV, some 80% of cells remained at G0/G1. In addition, there was an increased G2/M population when cells were treated with 4 μM EFV at both time points, from 4.6 to 16.1% at 24 h and 2.7 to 10% at 48 h (see Figures 3C,D). This however was not statistically significant.

#### FACS Analysis of LPV/r Treated MRC-5 Cells

At 24 and 48 h some 60–70% of the vehicle control cells were located in the G0/G1 phase of the cell- cycle. Relative to this, the percentage of cells in G0/G1 decreased with increased drug concentration at both 24 and 48 h, dropping to about 51–52% following treatment with 10 μM LPV/r and 16–23% following treatment with 32 μM LPV/r. However, at the highest concentration of LPV/r treatment (80 μM), the percentage of cells in G0/G1 increased in the range of 45–57%. The percentage of cells in S-phase increased from ~3% at 24 and 48 h in control cells to 65 and 72%, respectively, at 24 and 48 h, with the 32 μM LPV/r treatment. At 80 μM LPV/r, the percentage of cells synthesizing DNA decreased markedly to 26% at 24 h and 8% at 48 h. For G2/M phase, the proportion of cells increased marginally from about 30% to about 20% at 24 and 48 h, respectively, when treated with 10 μM LPV/r. At higher concentrations, the cell percentages decreased to below those at the levels of the control (see Figures 3E,F).

#### FACS Analysis of A549 Cells Treated With LPV/r

After drug treatment, the percentage of cells in G0/G1 decreased from 78 and 82% in vehicle control, to 19 and 24% when treated with 10 μM LPV/r. An increase in G2/M phase from 8 and 4% in vehicle control cells, to 42 and 22% when cells were treated with 10 μM LPV/r for the 24 and 48 h time points, respectively. When treated with 32 μM LPV/r this increased again to about 49 and 32%, at 24 and 48 h, respectively. At the highest concentration of LPV/r these percentages remaining in a similar range at both time periods. The relative stability of these proportions at the upper concentrations of LPV/r signifies an S-phase arrest. A sub-G0/G1 population was detected in response to 80 μM LPV/r, indicating cell-death (see Figures 3G,H).

#### Both EFV and LPV/r Alter the Cell-Cycle Stages

FACS analysis more precisely determined the effects of the ARV drugs, EFV, and LPV/r on cell-cycle stages. In summary, at low concentrations and at each time point, the ARVs effectively stimulated an increase in the percentage of cells in the G2/M phase in normal and cancerous cells. At higher concentrations, an S-phase arrest occurred, which is usually preceded by DNA damage. This results in cells with damaged DNA being unable to proceed to the G2 phase. Thus, it would seem that at higher concentrations LPV/r causes irreparable DNA damage, potentially leading to apoptosis. At the maximum ARV concentrations used here, cell viability was reduced, leading to the detection of a sub-G0/G1 cell population.

### The Effect of ARVs on Apoptosis, Figure 4

The ability of LPV/r to induce programmed cell death is demonstrated by LPV/r having a cytotoxic effect on both the normal MRC-5 and cancerous A549 cells; whereas EFV in comparison did not seem to predispose cells to apoptosis. Further to this, the demonstration of a sub-G0/G1 population after LPV/r treatment prompted additional investigation of the cytotoxic/apoptotic effects of LPV/r.

**Figure 4:**
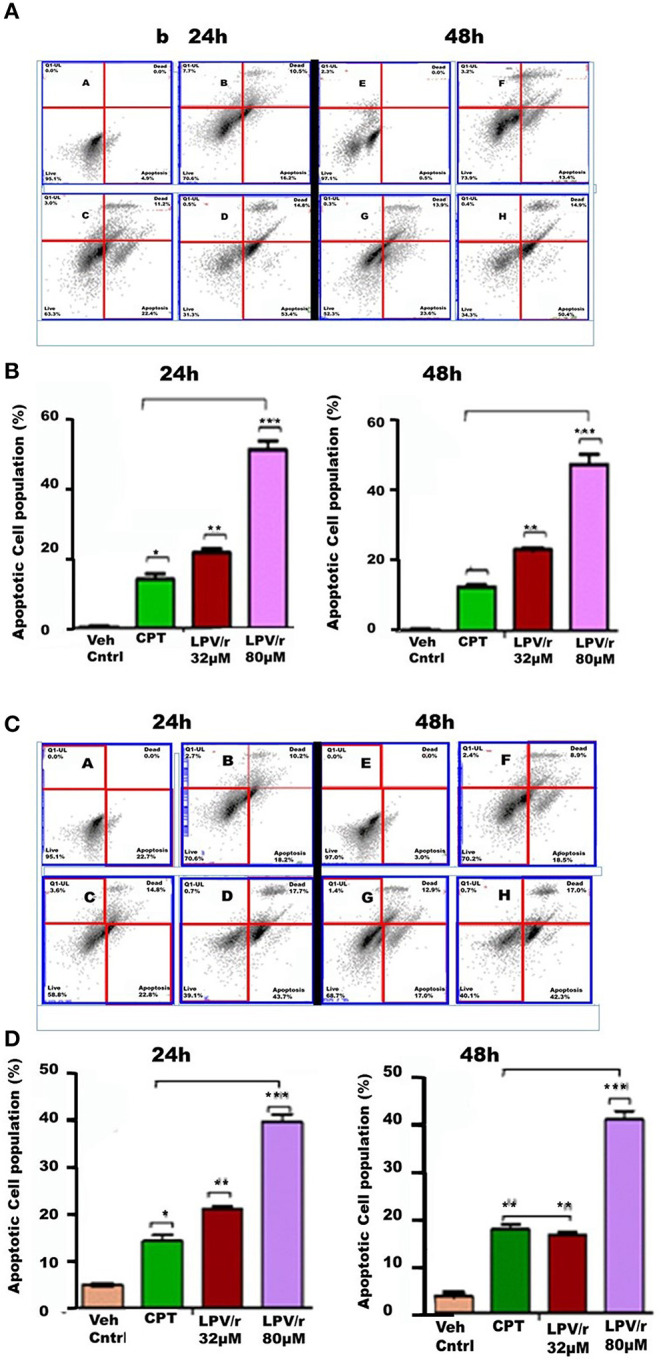
FACS apoptosis analysis of MRC-5 and A549 cells in response to LPV/r. **(A,B)** Show cytograms and bar graphs of MRC-5 cells treated with LPV/r, while **(C,D)** represent cytograms and bar graphs of LPV/r treated A549 cells. Error bars denote standard error of the means (SEM); *p < 0.05; **p < 0.01; ***p < 0.001. Results represent three independent experiments which were done three times independently.

#### Induction of Apoptosis by LPV/r

Following 32 and 80 μM LPV/r drug treatments, including Camptothecin (CPT) (50 μM) as positive control, FACS analysis using Annexin FITC and PI staining was used to quantify and analyse apoptosis in the lung cell lines. Control and treated cells were labeled with both Annexin FITC and PI. The control-unstained cells were used as a reference blank, the control- stained cells, the negative control, while Annexin-FITC single staining and PI single staining were used for compensation and setting up of quadrants. These results are represented in histograms and bar-graphs (Figure 4).

#### LPV/r Drug Treatment Induces Cell Death (Apoptosis and Necrosis) in a Dose Dependent Manner

LPV/r at both, of 32 and 80 μM, induced apoptotic effects on normal and cancerous lung cells, acting to increase the percentage of cells undergoing apoptosis with an increasing LPV/r concentration. However, with this a significant coupled cellular necrosis occurred in both MRC-5 and A549 cells. As represented in Figures 4A,B, treatment of MRC-5 cells with 32 μM LPV/r led to a slightly higher degree of apoptosis, compared to the CPT treated MRC-5 cells; whilst this effect was only evident at 24 h in A549 cells, Figures 4C,D. With 80 μM LPV/r treatment although there was a doubling in the percentage of cells undergoing apoptosis compared to 32 μM treated cells (Figure 4), necrotic cell death nevertheless, did not increase with increasing LPV/r concentrations.

## Discussion

The cellular responses to antiretroviral treatment (ART) were assessed in real time to quantitate cell proliferation and to effectively determine cellular response to the pharmacological treatments. The ARVs acted to decrease cell viability in a dose-dependent manner in both cell lines. Notably, however, the two-plasma level equivalent EFV concentrations increased cell proliferation, while only the lowest LPV/r treatment caused a proliferative increase. Moreover, the most physiologically relevant LPV/r dose resulted in growth arrest in lung cancer cells. Thus, depending on concentration and at specific window periods of treatment, both EFV and LPV/r can exert either pro- or anti-tumorigenic effects on cells. The cell-cycle is normally a tightly regulated process with multiple control points at different phases of cell growth, with the failure or improper functioning of these check points potentially leading to either abnormal cell proliferation or apoptosis. In association with increased cell proliferation, subsequent cell-cycle analyses showed a significant increase in S-phase in response to ARV treatments; with an apoptosis inducing effect of one of the ARVs (LPV/r). However, it was noted that besides apoptosis, LPV/r treatment additionally triggered necrotic cell death in a time-dependent manner.

To date, several studies including (23, 24) have revealed the cytotoxic effects of EFV against several cancer cells including colorectal and pancreatic cancer, but to our knowledge, no study yet has shown the anti-proliferative effects of EFV on lung epithelial cancer cells in relation to the primary lung fibroblast cells. Notably, our study demonstrates the anti-proliferative effects rather than the cytotoxic effects of EFV on lung cells, particularly against the A549 cancer cells and sparing the normal fibroblast MRC-5 cells, as Hecht et al. (23) demonstrated (23). Jin et al. (25) also revealed that EFV increased the expression of CASP3 and BAX, thereby reducing the proliferation of neuronal stem cells (25). EFV also causes morphological changes in cells. EFV has been shown to cause apoptosis in the Human Squamous Cell carcinoma from Uterine Cervix (HCS-2) cells and a change was observed in morphological features such as rounding-up of cells, retraction of filopodia, blebbing, and maintenance of plasma membrane integrity- characteristic features of apoptosis (26).

The protease inhibitor (PI) lopinavir is used for the treatment of HIV infections (27–35). Lopinavir has been shown to induce proteotoxic and oxidative stress, and also suppress NF-κB activity (36–38) The apoptotic and anti-tumor properties of LPV have been previously reported (39). Bissinger et al. (30) showed that LPV induced apoptosis in erythrocytes, accompanied by cell shrinkage and phospholipid scrambling (30). Okubo et al. (40) also showed the anti-proliferative properties of lopinavir/ritonavir (LPV/r) in combination against urological cancer cells. This study used 40/10 μM ratio of LPV/r over 48 h, and indicated that LPV/r treatment induced endoplasmic reticulum (ER) stress and kills urological cancer cells (40). Lopinavir was also shown to inhibit melanoma cell proliferation, induce morphological changes, apoptosis, and reactive oxygen species production, (41). A previous study revealed the anti-proliferative and cytotoxic effects of LPV/r at 20 μM) over 72 h in ovarian cancer. This was accompanied by G1 cell cycle arrest in ovarian cancer cells. LPV/r treatment in this cancer inhibited AKT signaling and this resulted in the inhibition of migration and invasion of ovarian cancer cells, and induction of apoptosis (42).

Based on these observations, it is proposed here that both EFV and LPV/r alter the cell-cycle progression of both normal and cancerous cells. In particular they lead to an arrest at the S-phase inhibiting further progression through the cycle, with LPV/r having the ability to inducing apoptosis. The apoptotic inducing properties of LPV/r merit further investigations not only as an ARV drug, but also as a potential anti-cancer treatment. However, a current limitation of LPV/r is its ability to not only kill tumor cells, but also to eliminate normal healthy cells. On the other hand, while an S-phase arrest is evident from both EFV and LPV/r treated cells, DNA damage usually precedes S-phase arrest. It follows then that both EFV and LPV/r could potentially be causing damage to the genomic DNA, with an arrest at S-phase, during which time there may be an attempt to either repair the damaged DNA or an induction of premature senescence, or even cell death. While the S-phase arrest is induced in the A549 lung cancer cells, it is also evident in the normal MRC-5 cells. This observation implicates both EFV and LPV/r as inducing stress on the DNA, with cells attempting to establish defense mechanisms by blocking the progression to G2/M phase. However, prolonged and constitutive stress effects of these ARVs on normal cells eventually exhaust the cells' repair mechanisms, and this may lead to uncontrolled cell proliferation and tumorigenesis. Furthermore, the cytotoxic effects of EFV on tumor cells such as colorectal cancers were shown by Hecht et al. (23), while primary fibroblast were unaffected. In addition, LPV/r's cytotoxic effects as a potential treatment for cancer was previously reviewed by Maksimovic-Ivanic et al. (43). The limitation of this study is the short exposure time (24–72 h) of lung cells to the ARVs, while in a clinical setting, patients on HAART have been exposed to these drugs for many years. In view of the double-edged properties of these drugs reported on in the present study, using patient samples may aid in a better understanding of these findings. The great potential of repositioning EFV and LPV/r for the treatment of cancer is of paramount significance, as the repurposing of current drugs provide economic benefit as well as helping to fulfill the need for new cancer treatments.

## Summary

**Figure 5:**
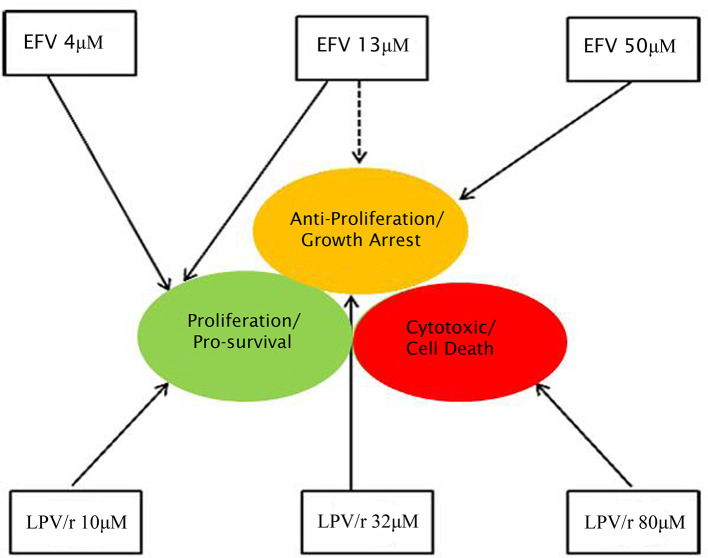
Diagrammatic representation of the effects of EFV and LPV/r at low and high doses. Both EFV and LPV/r exhibit pro-survival effects at low doses, while anti-proliferative and cytotoxic effects are observed at high doses. The solid arrows represent the effects of the drugs on cellular health, while the dashed line shows partial/dual effect. At a high dose, EFV is anti-proliferative, arresting cellular growth, while low doses favor survival modes, as also observed with low LPV/r dose. In contrast, moderate (plasma-level) and high LPV/r doses have anti-proliferative and cytotoxic properties on the cells.

A model summarizing the pro- and anti-proliferative effects of EFV and LPV/r is represented in Figure 5. In this model, treatment of A549 and MRC-5 cells with various concentrations of EFV and LPV/r leads to either proliferative effects with lower concentrations, or a growth arrest with higher EFV concentrations and mid-level concentrations of LPV/r. Finally, treatment with higher concentrations of LPV/r led to cytotoxic effects on both cell lines.

## Data Availability Statement

The raw data supporting the conclusions of this article will be made available on request from the corresponding author.

## Author Contributions

RM and CP conceived and initiated this project. All experiments described in this manuscript were performed by RM who then generated all figures of this paper. RM, CP, RH, and ZD all contributed to the writing of this paper. RH generated the schematic summary of this manuscript, while ZD further edited this manuscript. All authors contributed to the article and approved the submitted version.

## Conflict of Interest

The authors declare that the research was conducted in the absence of any commercial or financial relationships that could be construed as a potential conflict of interest.
